# An Integrative Analysis Revealing ZFHX4-AS1 as a Novel Prognostic Biomarker Correlated with Immune Infiltrates in Ovarian Cancer

**DOI:** 10.1155/2022/9912732

**Published:** 2022-06-26

**Authors:** Changchang Huang, Hongyin Cui, Xiaolin Lang, Fen Zhao

**Affiliations:** Department of Gynecology, First People's Hospital of Linping District, Hangzhou, Zhejiang, China

## Abstract

Ovarian cancer (OC) is the main cause of deaths worldwide in female reproductive system malignancies. Growing studies have indicated that eRNAs could regulate cellular activities in various tumors. Yet the potential roles of eRNAs in OC progression have not been elucidated. Thus, comprehensive assays were needed to screen the critical eRNAs and to explore their possible function in OC. We used Kaplan–Meier methods to identify survival-associated eRNAs in OC based on TCGA datasets. The levels of ZFHX4-AS1 were examined using TCGA datasets. Further exploration was carried out based on the following assays: clinical and survival assays, GO terms, and KEGG assays. TIMER was applied to delve into the relationships between ZFHX4-AS1 and tumor immune infiltration. In this research, we observed 71 survival-related eRNAs in OC patients. ZFHX4-AS1 was highly expressed in OC specimens and predicted a poor prognosis of OC patients. In addition, high ZFHX4-AS1 expression was positively related to the advanced stages of OC specimens. Multivariate assays revealed that ZFHX4-AS1 was an independent prognostic factor for overall survival of OC patients. KEGG analysis indicated that ZFHX4-AS1 may play a regulatory effect on TGF-beta signaling, PI3K-Akt signaling, and proteoglycans in cancer. The pan-cancer validation indicated that ZFHX4-AS1 was related to survival in eight tumors, namely, UCEC, STAD, SARC, OV, ACC, KICH, KIRC, and BLCA. The expression of ZFHX4-AS1 was correlated with the levels of B cells, T cell CD8+, neutrophil, macrophage, and myeloid dendritic cells. Simultaneously, ZFHX4-AS1 may be a prognostic biomarker and a distinctly immunotherapy-related eRNA in OC.

## 1. Introduction

Ovarian cancer (OC) is a common gynecological malignant tumor and a main cause of disease-related death in women worldwide [1]. In most cases, OC patients are only diagnosed in the latter stages of the disease because the disease's pathophysiology is difficult to detect [2, 3]. More than 70% of patients had OCs outside the ovaries, whereas only 30% had OCs in the pelvic or abdominal organs [4, 5]. In the early stages, the majority of patients show no symptoms at all, and the common symptoms of the patients in the late stage include ascites, pelvic lumps, abdominal distension, bellyache, and emaciation [6]. FIGO stage, histological types, and age have all been found to be prognostic indicators for OC [7]. However, developing precise markers for the prediction of therapy responses and long-term survivals for OC patients is crucial due to the significant degrees of heterogeneity of OC.

Enhancer RNAs (eRNAs) are a group of long noncoding RNAs derived from the enhancer regions [8]. The expression levels of eRNAs change in response to enhancer activity when transcription is bidirectional [9]. eRNAs have been recognized for more than 10 years but have not garnered adequate attention in the most recent reviews about the function of RNAs [10, 11]. A number of malignancies can be linked to the formation of eRNAs, which have recently been discovered thanks to the development and deployments of high-throughput sequencing and gene chip technologies [12, 13]. It is possible to employ eRNAs that are improperly expressed in cancer specimens as particular tumor biomarkers for early diagnosis and prognosis of cancer [14]. In addition, eRNAs can regulate chromatin modification or structure or alter gene transcription, splicing, and translation through direct interactions with protein, messenger RNAs, or DNA [15, 16]. Generally speaking, eRNAs play an important role in several biological processes in tumor developments, such as tumor growth, invasion, migration, stem cell reprogramming, and drug resistance [17, 18]. For instance, ADCY10P1 was found to be lowly expressed in OC and associated with poor overall survival. In vitro experiments revealed that the distinct upregulation of ADCY10P1 suppressed the proliferation, invasion, migration, and EMT phenotype of OC cell lines [19]. However, the potential roles of many eRNAs have not been investigated.

In this study, for the first time, we studied TCGA datasets and screened the survival-associated eRNAs in OC. Moreover, in order to examine the link between important eRNA and tumor-infiltrating immune cells, we used the TIMER method. In addition to shedding light on ZFHX4-AS1's critical role in OC progression, the findings in this study suggested a link between ZFHX4-AS1 and tumor-immune interactions.

## 2. Materials and Methods

### 2.1. Datasets

On November 20, 2021, we retrieved the RNA sequencing (RNA-seq) data of 378 OC patients and the accompanying clinical characteristics from TCGA database (https://portal.gdc.cancer.gov/repository). The GTEx database (https://xenabrowser.net/datapages/) was used to retrieve the RNA-seq data of 180 nontumor samples. The RNA-seq data and clinical information of the other cohort were collected from the GEO datasets (ID: GSE18520). The GSE18520 dataset was based on the GPL570 platform (HG-U133_Plus_2; Affymetrix Human Genome U133 Plus 2.0 Array) and contains 53 OC specimens and 10 nontumor specimens.

### 2.2. The Identification of Survival-Associated eRNAs in OC

The false discovery rate- (FDR-) adjusted *P* < 0.05 was applied as standard cut-off values for the identification of the survival-associated eRNAs by the use of Kaplan–Meier assays. All eRNAs which meet the standard were considered as survival-related eRNAs. According to the median expressions of each eRNA, the patients were divided into low- and high-expression groups. Between the two groups using *P* < 0.05, differences in survivals were compared.

### 2.3. Functional Enrichment Analysis

Genes in OC that had a significant association with the important eRNA were identified using Pearson's correlation analysis (cor > 0.4, and *P* < 0.001). Then, the Gene Ontology (GO) and Kyoto Encyclopedia of Genes and Genomes (KEGG) analyses based on the genes which were associated with the expression of the key eRNA were conducted by the “clusterProfiler” R package [20].

### 2.4. Verification in Pan-Cancer

Firstly, the R limma package was applied to gather the expressing data of ZFHX4-AS1 in pan-cancer, and then, the expressing matrix was coupled with pan-cancer survival data. ZFHX4-AS1 expression was utilized to classify the samples into high- and low-expression groups, and the survival differences between the two groups were compared using the Kaplan–Meier assays. *P* < 0.05 was considered statistically significant. A survival curve was finished for ZFHX4-AS1 in tumor that met the criteria.

### 2.5. TIMER Database Analysis

TIMER is a comprehensive resource for systematic analysis of immune infiltrates across diverse cancer types [21]. Immune infiltrates can be estimated using data from the TIMER database, which has 10,897 samples from 32 different kinds of tumors from TCGA. We studied ZFHX4-AS1 expression in various cancers and the connection of ZFHX4-AS1 expressions with the abundance of immune infiltrates. Additionally, correlation modules were used to investigate the relationship between ZFHX4-AS1 expressions and genetic biomarkers of tumor-infiltrating immune cells.

### 2.6. Statistical Analysis

All statistical analyses were conducted using R (version 3.6.0). Pearson's chi-square tests were executed for the comparison of categorical variables. Comparing survival rates between groups was done statistically using Kaplan–Meier curves and log-rank tests. Univariate and multivariate assays were utilized to further determine the prognostic values of eRNAs. *P* value < 0.05 was considered statistically significant.

## 3. Results

### 3.1. The Identification of Key eRNAs in OC Patients

**Figure 1:**
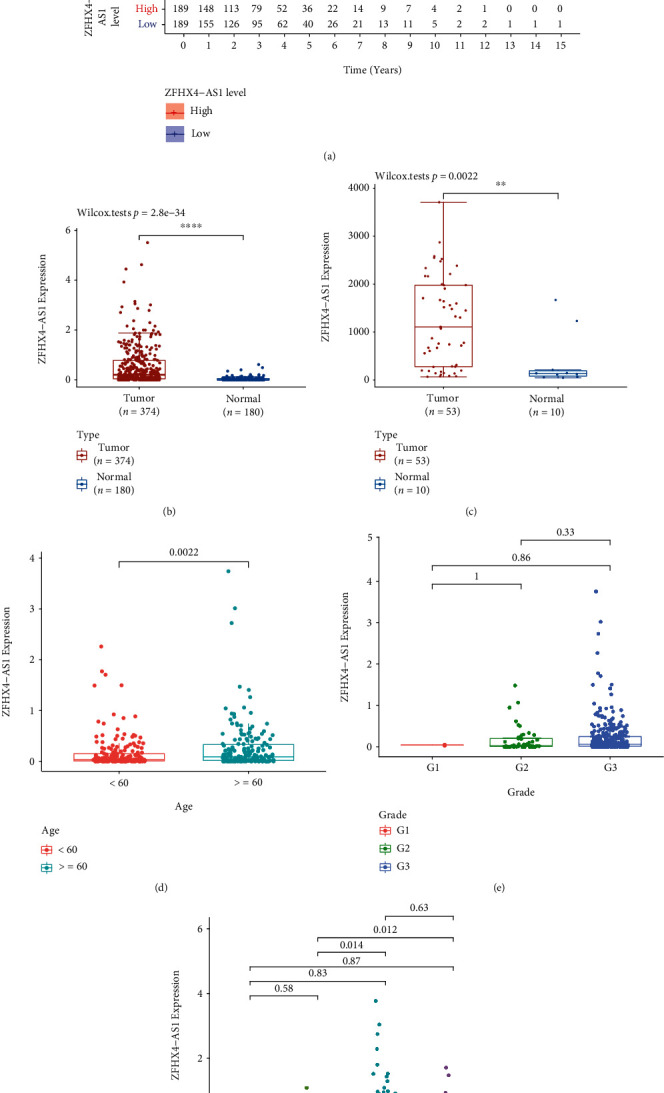
The expression of ZFHX4-AS1 in OC and clinical significance. (a) Kaplan–Meier survival curves for ZFHX4-AS1 based on TGCA datasets. (b) ZFHX4-AS1 expression in 374 OC specimens and 180 nontumor specimens. (c) The expression of ZFHX4-AS1 in 53 tumor samples and 10 nontumor samples from GSE18520. The relationships between ZFHX4-AS1 and clinical features, including (d) age, (e) grade, and (f) stage. ^∗∗∗∗^*P* < 0.0001 and ^∗∗^*P* < 0.01.

Firstly, we performed the Kaplan–Meier method to screen the survival-related eRNAs using *P* < 0.05 via analyzing TCGA datasets. We observed 71 survival-related eRNAs in OC patients (Table S1). Then, our attention focused on ZFHX4-AS1 which was rarely reported in tumors (Figure 1(a)). Importantly, our team observed that ZFHX4-AS1 expressions were distinctly increased in OC specimens compared with nontumor specimens via analyzing TCGA and GTEx database (Figure 1(b)). Moreover, we analyzed GSE18520 and also confirmed that ZFHX4-AS1 was a highly expressed eRNA in OC (Figure 1(c)). Overall, ZFHX4-AS1 was found to be a new regulator in the progression of OC, according to our findings.

### 3.2. The Clinical Significance of ZFHX4-AS1 Expression in OC Patients

**Figure 2:**
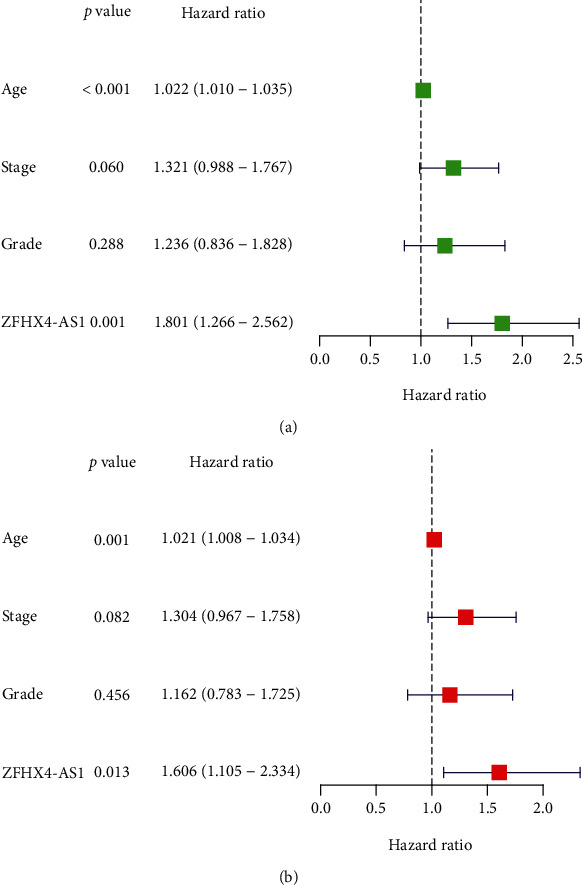
(a) Univariate and (b) multivariate assays were applied to further explore the prognostic value of ZFHX4-AS1 in OC patients.

Then, OC patients' ZFHX4-AS1 expression was examined in relation to their clinical characteristics. As shown in Figure 1(d), we observed that high ZFHX4-AS1 expressions were related to age of OC patients (*P* = 0.0022). However, we did not observe a distinct association between ZFHX4-AS1 expression and grade of OC patients (Figure 1(e)). Importantly, ZFHX4-AS1 levels were higher in advanced OC specimens than they were in early OC specimens (Figure 1(f)). Univariate Cox regression analysis showed that overall survival was strongly influenced by age (HR = 1.022, *P* < 0.001) and ZFHX4-AS1 expression (HR = 1.801, *P* = 0.001) (Figure 2(a)). Also, multivariate assays revealed that ZFHX4-AS1 was an independent prognostic factor for overall survival (HR = 1.606, 95% CI: 1.105-2.334, *P* = 0.013, Figure 2(b)). Our finding suggested that ZFHX4-AS1 may be involved in clinical progression of OC and may be a novel prognostic biomarker for OC patients.

### 3.3. Functional Analyses Based on the Genes Associated with the Expression of ZFHX4-AS1 in OC

**Figure 3:**
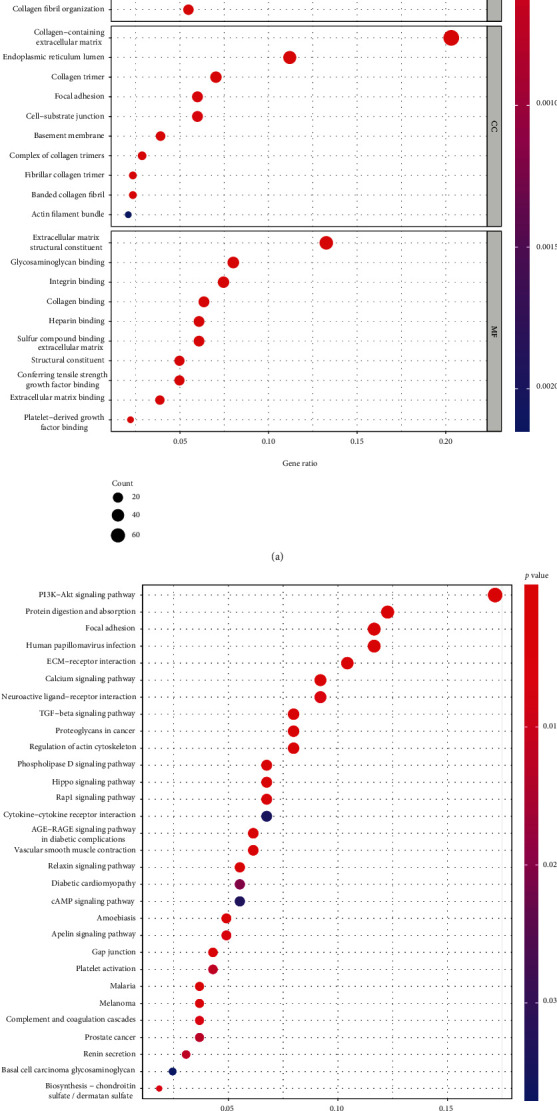
(a) Gene Ontology enrichment analysis. (b) The top 30 enriched KEGG pathways.

The results of Pearson's correlation analysis confirmed 457 genes which presented a distinct relationship with ZFHX4-AS1. Based on GO and KEGG assays of 457 genes, the biological investigation of these genes was able to be revealed.  Figure 3(a) summarizes the top 10 phrases for cellular component (CC), biological process (BP), and molecular function (MF). In BP, the terms were mainly associated with external encapsulating structure organization, extracellular matrix organization, and extracellular structure organization. In CC, they were related to collagen-containing extracellular matrix, endoplasmic reticulum lumen, and collagen trimer. In MF, term enrichment mainly involved extracellular matrix structural constituent, glycosaminoglycan binding, and integrin binding.  Figure 3(b) summarizes the top 30 routes. The results of KEGG assays revealed that the most distinctly enriched biological processes included human papillomavirus infection, TGF-beta signaling, focal adhesion, protein digestion and absorption, PI3K-Akt signaling, and proteoglycans in cancer.

### 3.4. Pan-Cancer Verification

**Figure 4:**
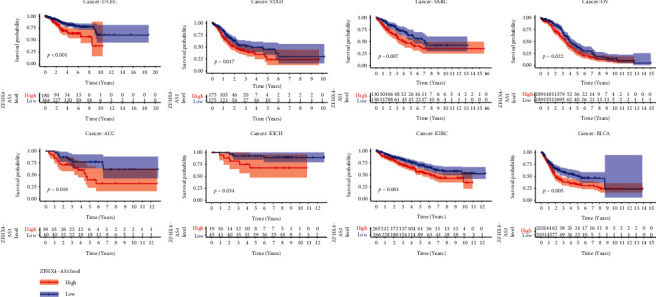
Kaplan–Meier assays for ZFHX4-AS1 in pan-cancer.

Survival and correlation analyses were carried out to assess the predictive significance of our chosen eRNA in pan-cancer. Our team showed that ZFHX4-AS1 was related to survivals in eight tumors, namely, uterine corpus endometrial carcinoma (UCEC), stomach adenocarcinoma (STAD), sarcoma (SARC), ovarian serous cystadenocarcinoma (OV), adrenocortical carcinoma (ACC), kidney chromophobe (KICH), kidney renal clear cell carcinoma (KIRC), and bladder urothelial carcinoma (BLCA). The survival curves for ZFHX4-AS1 in these eight tumors are shown in Figure 4. Our findings highlighted the important roles of ZFHX4-AS1 in tumor progression.

### 3.5. ZFHX4-AS1 Expression Correlates with Immune Cell Infiltration in OC

**Figure 5:**
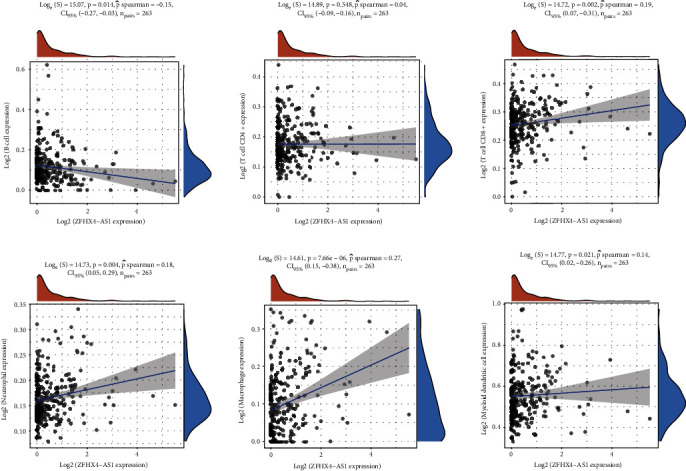
Correlations of ZFHX4-AS1 expressions with the levels of immune infiltration in OC.

Tumor metastatic status was found to be an independent predictor of prognosis and survival in patients with OC in the Kaplan–Meier Plotter database analysis [22]. Therefore, we analyzed whether ZFHX4-AS1 expressions were related to the levels of immune cell invasion in OC. Analysis of ZFHX4-AS1 expression and immune infiltrating cells in OC was carried out using the TIMER database. The results showed that the expression of ZFHX4-AS1 was negatively correlated with the expression levels of B cells, while positively related to the levels of T cell CD8+, neutrophil, macrophage, and myeloid dendritic cells (Figure 5). Our findings suggested that ZFHX4-AS1 may play an important role in the immune microenvironment.

## 4. Discussion

Experiments in recent years have established that the growth, division, metastasis, and invasion of OC cells are controlled by eRNAs [23, 24]. It is possible that the abnormal expression of some lncRNAs in OC will provide useful information for the diagnosis and treatment of patients, as well as their prognosis [25, 26]. However, the investigation of eRNAs for OC is still in its infancy compared to miRNAs. Therefore, further research into eRNAs in OC is required. We have been prompted by previous research to look for eRNAs connected with OC prognosis.

In this study, our team identified 71 prognostic eRNAs in OC patients via analyzing TCGA datasets. Among them, we focused on ZFHX4-AS1 which was related to poor outcome of OC patients. The studies on ZFHX4-AS1 function in tumors were rarely reported. Li et al. firstly reported that ZFHX4-AS1 was distinctly overexpressed in breast cancer and its silence suppressed the proliferation, invasion, and migration, while promoted cell apoptosis by the use of suppressing the Hippo signaling pathway. The above findings suggested ZFHX4-AS1 as an oncogene in breast cancer [27]. Hence, we also found that ZFHX4-AS1 expressions were distinctly upregulated in OC, which was consistent with its trend in breast cancer. Besides, we found that high ZFHX4-AS1 expressions were related to age and clinical stage of OC patients. More importantly, multivariate assays revealed that ZFHX4-AS1 was an independent prognostic factor for overall survival of OC patients, highlighting its potential use as a marker for the clinical outcome of OC patients. In addition, we performed KEGG assays which revealed that ZFHX4-AS1 could exhibit a regulatory function on the clinical outcomes of OC patients through PI3K-Akt signaling pathway, protein digestion and absorption, focal adhesion, human papillomavirus infection, TGF-beta signaling pathway, and proteoglycans in cancer. The above pathways have been confirmed to be involved in the progression of tumors [28, 29]. Our findings suggested that ZFHX4-AS1 may be an important regulator in tumor progression. In pan-cancer studies, our team confirmed that ZFHX4-AS1 was related to long-term survivals in eight types of tumors (UCEC, STAD, SARC, OV, ACC, KICH, KIRC, and BLCA). Overall, the above findings suggested that ZFHX4-AS1 could be applied as an independent predictor of OC.

Cancer patients' prognosis and immunotherapeutic effectiveness can be predicted by tumor-infiltrating lymphocytes (TILs) in the tumor microenvironment (TME) [30, 31]. Researches showed that eRNAs were linked to TILs and play a critical function in the TME process [32, 33]. Our finding unraveled that the expression of ZFHX4-AS1 was negatively correlated with the expression levels of B cells, while positively correlated with the expressions of T cell CD8+, neutrophil, macrophage, and myeloid dendritic cells. Cancer immunotherapy's effector cells, CD8+ T lymphocytes, are widely known. CD8+ T lymphocytes have traditionally been stimulated to kill malignancies through Fas-Fas ligand pathways or the release of perforin-granzyme [34]. According to the findings of the two most recent research, immunotherapy-activated CD8+ T lymphocytes can cause ferroptosis in tumor cells through boosting lipid peroxidation [35, 36]. Our findings further revealed that ZFHX4-AS1 was the key molecule for bridging immunotherapy.

Several limitations should be resolved. Firstly, selection and recall bias are inescapable because of the retrospective design and in spite of strong inclusive and exclusive criteria. Secondly, it was not possible to determine the prognostic values of chemotherapy and radiation in the current investigation due to a lack of complete regimens. Thirdly, ZFHX4-AS1's role in cancer has been revealed by bioinformatic analysis, but further studies in vitro and in vivo were needed for further validation of our findings and the development of clinical applications. In order to understand ZFHX4-AS1's molecular and cellular functions, further mechanistic investigations are needed.

## 5. Conclusion

Our study screened 71 survival-related eRNAs in OC patients and validated that ZFHX4-AS1 was highly increased in OC, and its expressions were distinctly related to age and clinical stage. Patients with high ZFHX4-AS1 expressions showed a distinct decrease in OS, and ZFHX4-AS1 could be used as a biomarker for the prognosis of OC. In addition, ZFHX4-AS1 could be applied in the development of new-targeted drugs for immunotherapy. Our results may provide novel clues for the studies of molecular mechanisms involved in ZFHX4-AS1 function in OC.

## Data Availability

The datasets used and/or analyzed during the current study are available from the corresponding author upon reasonable request.

## Supplementary Materials

Supplementary Materials Table S1: the list of all survival-related eRNAs in OC.
