# Supplementary material for: An Integrative Analysis Revealing ZFHX4-AS1 as a Novel Prognostic Biomarker Correlated with Immune Infiltrates in Ovarian Cancer
Source: J Immunol Res. 2022 Jun 26;2022:9912732. doi: 10.1155/2022/9912732 (PMC9251081; doi:10.1155/2022/9912732)
Supplement: Supplementary Materials — Table S1: the list of all survival-related eRNAs in OC. [file 9912732.f1.docx]

Table S1 The list of all survival-related eRNAs in OC.

| gene | KM |
| --- | --- |
| ITFG2-AS1 | 0.021636 |
| AC082651.3 | 0.045548 |
| CTBP1-DT | 0.03349 |
| IFNG-AS1 | 0.011094 |
| LINC00487 | 0.041507 |
| GAS1RR | 0.045424 |
| LINC01277 | 0.039724 |
| LNC-LBCS | 0.032949 |
| MRLN | 0.0123 |
| LINC02754 | 0.008021 |
| LINC02384 | 0.003561 |
| HCP5 | 0.036208 |
| SLC44A3-AS1 | 0.046547 |
| STEAP1B | 0.024974 |
| LINC02487 | 0.004712 |
| LINC01731 | 0.004845 |
| AC104653.1 | 0.036763 |
| LINC00404 | 0.049432 |
| AL121972.1 | 0.011461 |
| AC139491.2 | 0.043909 |
| DARS-AS1 | 0.003716 |
| LINC01381 | 0.028802 |
| AC005515.1 | 0.020965 |
| AC109492.1 | 0.042336 |
| LINC01122 | 0.019781 |
| LINC02525 | 0.031659 |
| AC119677.1 | 0.021924 |
| BSN-DT | 0.047173 |
| CALML3-AS1 | 0.034364 |
| AC007389.1 | 0.020366 |
| AC141930.1 | 0.04892 |
| AC069120.1 | 0.033795 |
| AL391845.1 | 0.023218 |
| AC004540.1 | 0.02652 |
| AL391840.1 | 0.042188 |
| ZFHX4-AS1 | 0.021598 |
| AC022613.1 | 0.03514 |
| LINC02777 | 0.002196 |
| HAGLROS | 0.013108 |
| AC022784.1 | 0.043687 |
| AL354761.1 | 0.000431 |
| Z93930.2 | 0.045104 |
| MRPS31P5 | 0.008664 |
| LINC01088 | 0.046962 |
| AL359636.2 | 0.048415 |
| LINC01270 | 0.031932 |
| AC067930.2 | 0.006717 |
| LINC00467 | 0.003026 |
| FLVCR1-DT | 0.011175 |
| MAL2 | 0.006596 |
| LINC00582 | 0.00274 |
| AP002754.1 | 0.022768 |
| AL138787.1 | 0.037747 |
| LINC02006 | 0.005847 |
| LINC02365 | 0.045914 |
| PAAF1 | 0.024575 |
| AC092490.1 | 0.025348 |
| MAFA-AS1 | 0.025513 |
| LINC00665 | 0.011964 |
| FOXP4-AS1 | 0.005134 |
| LINC00996 | 0.013296 |
| HIVEP2 | 0.043216 |
| LINC01176 | 0.035666 |
| AC096759.1 | 0.00202 |
| LINC02696 | 0.042344 |
| LINC01252 | 0.040134 |
| Z94721.1 | 0.04869 |
| AC021028.1 | 0.03821 |
| SLC2A1-AS1 | 0.037372 |
| AC007848.1 | 0.048871 |
| UBA6-AS1 | 0.007742 |
